# Sound Symbolism in the Languages of Australia

**DOI:** 10.1371/journal.pone.0092852

**Published:** 2014-04-21

**Authors:** Hannah Haynie, Claire Bowern, Hannah LaPalombara

**Affiliations:** Department of Linguistics, Yale University, New Haven, Connecticut, United States of America; University of Bristol, United Kingdom

## Abstract

The notion that linguistic forms and meanings are related only by convention and not by any direct relationship between sounds and semantic concepts is a foundational principle of modern linguistics. Though the principle generally holds across the lexicon, systematic exceptions have been identified. These “sound symbolic” forms have been identified in lexical items and linguistic processes in many individual languages. This paper examines sound symbolism in the languages of Australia. We conduct a statistical investigation of the evidence for several common patterns of sound symbolism, using data from a sample of 120 languages. The patterns examined here include the association of meanings denoting “smallness” or “nearness” with front vowels or palatal consonants, and the association of meanings denoting “largeness” or “distance” with back vowels or velar consonants. Our results provide evidence for the expected associations of vowels and consonants with meanings of “smallness” and “proximity” in Australian languages. However, the patterns uncovered in this region are more complicated than predicted. Several sound-meaning relationships are only significant for segments in prominent positions in the word, and the prevailing mapping between vowel quality and magnitude meaning cannot be characterized by a simple link between gradients of magnitude and vowel F2, contrary to the claims of previous studies.

## Introduction

The notion that linguistic forms and meanings are related only by convention and not by any systematic relationship between sounds and semantic concepts, articulated by de Saussure [1] as the *arbitrariness of the sign*, is a foundational principle of modern linguistics. Though this principle generally holds across the lexicon, exceptions to arbitrariness have been identified. These iconic and onomatopoetic forms, in contrast with most other linguistic material, are characterized by a symbolic, non-arbitrary relationship between the form of linguistic representations and the meanings they convey.

Sound symbolism is defined as “the direct linkage between sound and meaning” ([8], page 1), which we interpret to mean a non-arbitrary sound-meaning association, or the greater than chance occurrence of a particular phoneme in a particular semantic category. Sound symbolism has been identified in lexical items and linguistic processes in many individual languages [2]–[6], and comparative studies have led to generalizations about the basic patterns of sound symbolism that have some cross-linguistic basis [7]–[10]. However, few systematic studies have been undertaken to examine the extent to which common patterns of sound iconicity are found in individual languages and across language areas, and there are no comparative studies for Australian Indigenous languages.

This paper examines sound symbolism in the languages of Australia, using data from a sample of 120 languages. We conduct a statistical investigation of the evidence for several patterns of sound symbolism in individual languages and across this continent more generally. The patterns examined here include the traditional association of meanings denoting “smallness” or “nearness” with front vowels or palatal consonants, and the association of meanings denoting “largeness” or “distance” with back vowels, as identified by previous studies. We also test for a proposed association between velar consonants with “largeness”/“distance” meanings, as well as testing for symbolic patterns among classes of sounds not explicitly associated with our hypotheses, including high and low vowels, lateral consonants, and labial consonants. Lastly, we also examine correlations between patterns identified as sound symbolic, but with the opposite meaning categories (for example, front vowels and meanings associated with “largeness” rather than with “smallness”). Our results provide evidence for the expected associations of vowels and consonants with meanings of “smallness” and “proximity” in Australian languages. However, the patterns uncovered in this region are more complicated than predicted. Several sound-meaning relationships are only significant for segments in prominent positions in the word, and the prevailing mapping between vowel quality and magnitude meaning cannot be characterized by a simple link between gradients of magnitude and vowel F2, contrary to the claims of previous studies.

### Sound Symbolism and its Motivations

Several different types of symbolism are found in language, motivated by both cognitive and communicative factors. Variation in the nature of the form-meaning relationships that define these categories predisposes certain types of sound symbolism to be exhibited through language-specific phenomena, or to occur in very specific semantic or pragmatic contexts. Specific phonemes or phoneme clusters, for example, can become associated with particular semantics through the development of phonosemantic conventions. The association of the cluster /gl/ with meanings of luminosity in English (e.g. “glitter”, “glisten”, “glow”, “glimmer”) exemplifies this conventional sound symbolism. Though this pattern may be shared across closely related languages, it is unlikely to co-occur among unrelated languages. Analyses of these sub-morphemic sound-meaning correspondences tend to focus on language-specific metaphor [11] or their function in language processing [12], rather than any universal tendencies based on phonetic iconicity.

Other types of symbolism are attested more robustly in cross-linguistic data, but are restricted to specific semantic domains or pragmatic contexts. For example, the tendency for the names of body parts involved in articulation to include sounds involving those articulators, noted by Urban [13], exploits a natural link between the body and language. Another common form of sound symbolism uses sounds or intonational patterns to express emotional or physical states, as in Hinton et al.'s [8] corporeal sound symbolism, which includes phenomena like speaking with raised pitch when frightened, or even involuntary noises like coughing. In both of these types of symbolism, cross-linguistic resemblances in a very specific domain are derived from human anatomy or general communicative principles.

Other cross-linguistic sound symbolic patterns arise through the use of speech sounds to imitate environmental noises. Onomatopoetic forms such as animal sounds (“squawk”), machine noises, and certain motion noises (“whoosh” or “bang”) receive their forms through the imitation of sounds that occur outside of language. Like corporeal symbolism, iconicity of this type can create cross-linguistic similarities. However, convergence in onomatopoetic forms in unrelated languages can be traced back to the extralinguistic acoustic inputs on which they are modeled, rather than any communicative function of the speech sounds themselves.

The debate regarding motivations for sound symbolism and the universality of various patterns of sound-meaning linkage centers on a further type of sound symbolism. *Synesthetic sound symbolism*, as Hinton et al. [8] have called this category of iconicity, associates sounds or classes of sounds with properties of items in the world. The classic example of synesthetic sound symbolism is the use of contrasting sounds to represent variation in size of an object (known as magnitude sound symbolism), though properties like movement, shape, and color can also be expressed through similar patterns. For example, in Ewe, the word for ‘small’ is *kítsíkítsí*, with high front vowels and high tones, while the word for ‘large’ is *gbàgbàgbà*, with back vowels and low tone [9]. French *petit* ‘small’ versus *gros* ‘large’ shows the same vowel pattern. Synesthetic sound symbolism has been examined both cross-linguistically [7], [14], [15] and experimentally [16], [17], and while the results of these studies present conflicting evidence about the nature of synesthetic sound symbolism and the universality of sound-meaning mappings, there is robust support for the use of synesthetic sound symbolism to encode magnitude contrasts in a wide variety of languages. We focus here solely on this type of sound symbolism.

Debate in this area of research has centered on the proposal that an association between acoustic frequency and certain meanings (e.g. “smallness”) predisposes certain classes of sounds to be used in the expression of these meanings. This association could be either innate or experience-based. Several different types of accoustic features have been associated with synesthetic symbolism, including the duration of sounds, formant frequency values, pitch contours, and loudness. Perhaps the most frequently discussed form of synesthetic sound symbolism is the use of pitch to convey certain linguistic and social meanings. Ohala [18] relates sound symbolic uses of fundamental (F0) frequency in human speech to a basic pattern in animal communication that associates high frequencies with submission and lower frequencies with dominance, suggesting that these communicative uses of frequency are innate to humans as well as many other species. Ohala relates this “frequency code” to the commonly observed correlation between words with connotations of smallness and high freqency sounds, and the correlation between connotations of largeness and low frequencies.

Though the universality of acoustic frequency-based patterns of phonetic iconicity has been debated [7], [14], [19]–[21], many studies have noted an association between high acoustic frequencies and smallness meanings, which generally surfaces in the form of palatal (or palatalized) consonants and high, front vowels in words with such meanings [7], [14], [18]. For example, Ultan (p 531) quotes an example from the Native American isolate Karuk, where *iθári**ˑ**p* ‘fir’ contrasts with *it**ʃ**áni**ˑ**pit**ʃ*** ‘little fir’, with the latter showing palatal affricates which are associated with small-size sound symbolism. Alternative explanations place the iconicity in the realm of articulation, as a direct relationship between the size of the space between the tongue body and the palate and the acoustic energy associated with such articulation [22], [23]. Most studies, however, characterize magnitude symbolism in terms of acoustic frequency either instead of, or in addition to, these articulatory parameters. Frontness or F2 is most commonly cited as being responsible for the overall perception of vowel pitch [15], [16], [20]. For some phoneme classes, articulatory and acoustic associations coincide. For example, high front vowels have higher inherent pitch, smaller closure, shorter inherent duration, and higher F2 frequency than low back vowels [24], [25].

The reasoning described here does not exclusively pick out palatal obstruents and front vowels as candidates for iconic markers of magnitude concepts, however. For example, back vowels could be argued to belong to the “small” category because their articulation involves backing the tongue body and compressing the area of the velo-pharyngeal region (see further [24], especially p 261ff). In fact, magnitude sound symbolism potentially suffers from the problem that Roberts and Winters [26] discuss with respect to correlations between linguistic features and sociological or cultural ones; namely, that there are often multiple (more or less plausible) post-hoc explanations for correlations that are difficult to test rigorously. We recognize this problem. Others [7], [14], [19]–[21] have grounded explanations for these tendencies in universal acoustic and articulatory properties of the speech signal. However, because there are so many potential ways in which a “frequency code” might map onto magnitude sound symbolic categories, motivating unique phoneme classes is impossible. Our concern here is therefore to test the sound symbolic correlations among Australian languages that have been repeatedly identified (as discussed above and in the following section) with languages from other parts of the world.

### Evidence for Magnitude-related Sound Symbolism

The basic pattern of magnitude-related sound symbolism was probed in the early twentieth century by Sapir [10] and Newman [16]. These studies investigated preferences in invented word names for small/large pairs of items and found that subjects preferred to associate higher-frequency vowels (e.g. [i]) with the smaller member of a contrastive pair and lower-frequency vowels (e.g. [**ɑ**]) with the larger item. These early experiments have since been questioned on methodological grounds, as the stimuli they used forced subjects to associate sounds with a contrast, and the experiment design may have made the expected associations clear to study participants [17], [27]. A more sophisticated experiment by Thompson and Estes [17] also used name-object matching tasks to test whether the preferred names for objects of graded sizes demonstrated graded phonetic symbolism. However, these more nuanced results reaffirm the relationship between frequency and size, and further suggest that magnitude sound symbolism may be gradient in nature, rather than dichotomous, as earlier studies suggested. Shinohara and Kawahara [28] conducted a similar experiment among speakers of Chinese, English, Japanese, and Korean, requiring participants to guess the size of the referent of invented words. Their results demonstrate a clear difference in the backness of vowels associated with smallness versus largeness among speakers of all four languages, and positive but less straightforward link between smallness and vowel height, particularly in Chinese and Japanese.

The question of universality of sound symbolic patterns has also stimulated a number of cross-linguistic studies of magnitude symbolism. Early in the twentieth century, Jespersen [19] collected a number of examples of size-related words whose phonetic forms follow the frequency-related pattern noted above and exemplified by Ewe and French. Subsequent studies expanded upon Jespersen's Indo-European-heavy sample and looked more systematically at cross-linguistic patterns of magnitude-related sound symbolism. Ultan's [7] survey of sound symbolism in 136 languages included not only size and distance categories, but also several semantic categories that could be considered physical or metaphorical extensions of a size parameter (e.g. duration, grammatical distance). Though the sample used by Ultan was skewed by the inclusion of a large number of Native American languages, he found that the overall incidence of size symbolism was low; it was found in only 38 of his 136 sample languages. Somewhat more robust evidence is presented for distance symbolism (found in 46 sample languages). Yet among the languages that do exhibit size symbolism, he found that vowel *ablaut*, or sound alternation associated with morphological function, was a common strategy for expressing diminutive meaning, with front vowels being the predominant phonological category associated with diminutive meaning. Consonant ablaut, in contrast, he describes as “a complex of universal types due to its extremely localized distribution,” though he notes that palatal/fronted consonants, manner/degree of closure, and glottalization are commonly associated with diminutive meanings ([7], page 554). This thus implies that although sound symbolism might affect only a small part of the lexicon, it is robustly attested.

More recently, cross-linguistic studies of symbolism have focused on proximity/distance, rather than the broad array of magnitude symbolic meanings that Ultan surveyed. Woodworth [29] demonstrated support for the link between vowel F2 frequency and distance in a survey of demonstrative pronouns and locative adverbials. Half of the 26 languages in her sample exhibited higher F2 of vowels in proximal forms than distal forms, consistent with the frequency code hypothesis outlined by Ohala [18]. Traunmüller's [15] survey of deictic forms was couched in terms of pragmatic motivations for sound symbolism; nevertheless, the 37 proximal/distal demonstrative pronoun pairs he surveys show a striking consistency with the predictions of the frequency hypothesis and the scale of vowel magnitude symbolism developed by Newman [16]. A full 32 of the examples presented by Traunmüller are counted as supporting the symbolism hypothesis, and the percentages of proximal and distal forms that contain each vowel fit quite well along a cline [i, e, a, o, u] of vowel magnitude symbolism.

Existing literature on size and distance sound symbolism points toward an association between high frequency sounds and small/proximal meanings, in opposition with lower frequency sounds associated with larger/more distant meanings [7], [10], [15], [18], [29]. Some, such as Ohala [18], believe this frequency association to be universal or even innate, yet others argue against the universalist view. Bauer [14], for example, draws on a comparison of augmentative and diminutive morphology in a sample of 50 languages to argue against the innate frequency hypothesis that scholars like Ohala [18] have proposed. Her small dataset shows roughly even occurrences of high vowels, front vowels, and palatal consonants in diminutive and augmentative morphemes. A handful of authors have pointed out direct counterexamples to proposed universals of magnitude symbolism [3], [21]. Diffloth's examples from the Mon-Khmer language Bahnar serve as an anecdotal counterexample to the possibility that vowel height is universally linked to size sound symbolism, but this does not bear on hypotheses that link magnitude meanings to the F2 (or backness) of vowels. A further apparent counterexample is Korean [3], where lower vowels are associated with small meanings. Compare, for example, the pair *p^h^u**ŋ**t**ʌŋ*** ‘splash (of a large object)’ and *p^h^o**ŋ**ta**ŋ*** ‘splash (of a small object)’ ([3], page 437). However, as Ohala [9] points out, it is unclear whether the Korean pattern is an example of magnitude sound symbolism, or simply a case of grammatically marked intensification.

Independent of the question of universal motivation, the occurrence of sound symbolism in the world's languages has been claimed to be influenced by areality and borrowing. The languages in the Ultan [7] sample that exhibit sound symbolism, for example, are predominantly Native American languages, though they belong to a number of language families. Areality in North American sound symbolism, and in particular diminutive consonant patterns, was also noted by Nichols [30], who found specific types of diminutive consonant shifts distributed in geographic clusters in this region, suggesting spread through borrowing. Thus in summary, from the existing literature we find widespread evidence for sound symbolism in individual languages, but conflicting views as to its manifestations.

None of the previous cross-linguistic studies of sound symbolism have utilized data from Australian languages to any extent. This study of sound symbolism in Australian languages provides further evidence for the relationship between frequency and magnitude, and bears on several of the unresolved questions in the arena of size sound symbolism. If the association between front vowels or palatal consonants and small meanings is universal, for example, we would expect to find these sounds in words with “small” meanings in Australian languages.

The current study investigates phonological associations with magnitude generally, as well as two subtypes of magnitude symbolism, using the same sample of languages. To do so we test specific hypotheses regarding symbolic relationships between natural classes of sounds and magnitude-related meanings, which are based upon the literature discussed in this section. The ability to compare these subdomains of magnitude symbolism across a single language sample allows us to test whether the dominance of distance symbolism in Ultan's America-skewed sample holds for Australia or whether there are significant areal differences in these types of symbolism. Finally, we are able to quantify the evidence for symbolism on a language-by-language basis and by doing so look for trends in genealogically related languages as well as the sorts of areal patterns that Nichols [30] and Ultan [7] have identified in North America, and Jespersen [19] for Indo-European.

### Predictions of existing magnitude sound symbolism literature

The conclusions of experimental and cross-linguistic studies that have investigated size and distance sound symbolism make several predictions about the sound-meaning patterns we expect to find in Australian languages. The most frequently cited phonetic correlate of magnitude symbolism is the backness of vowels, or F2 in acoustic terms. Hinton et al.'s formulation of the “frequency code” explicitly associates this hypothesis with the claim that “vowels with high second formants … are associated with high frequency sounds” ([8], page 10). The F2 patterns reported by Shinohara and Kawahara, Fischer-Jorgensen, Thompson and Estes, and by Woodworth for distance meanings [17], [20], [28], [29] would also predict that front vowels are more likely to occur in forms for “smallness” and “proximity” meanings, while back vowels are more likely to occur in “largeness” and “distance” meanings.

Several studies make less clear predictions about the correlation of magnitude meanings with either vowel “backness” (F2) or vowel height (F1). Ultan finds high front vowels to be associated with diminutive categories, and while he does not present a definitive conclusion regarding the roles of height and backness in that symbolic relationship, he entertains the idea that F2 may be the relevant acoustic parameter in this pattern ([7], page 545). Shinohara and Kawahara also report an association between vowel height and magnitude, though this pattern is not as strongly supported as their findings regarding backness [28]. Newman [16] presents a vowel scale that does not neatly correspond to height and backness, and several subsequent papers also represent the symbolic magnitude of a vowel in terms of a cline. Though Newman's cline does not exactly correspond to F2, front vowels tend to fall on the “small” side of th spectrum, with /i/ at the far end, while back vowels tend to fall on the “large” side of the spectrum, with /u/ at the other extreme. Thus, while these works predict the involvement of both height and backness in vowel magnitude symbolism, they all make relatively stronger cases for the involvement of backness.

Predictions regarding consonants are murkier. Hinton et al.'s statement of the “frequency code” only claims that “high frequency consonants” and “low frequency consonants” in general will be associated with “small”/“proximal” and “large”/“distant” meanings respectively ([8], page 10). Interpretations of “high acoustic frequency” in the consonant domain vary quite substantially. Ultan's link between consonant fronting and diminutive meanings, if interpreted as evidence of a universal tendency, would predict that consonants with a forward place of articulation would be more likely to occur in “small”/“proximal” meanings than similar consonants with a farther back place of articulation. Experimental studies have tended to focus on voicing as the phonetic correlate of magnitude symbolism in consonants [17], [28]. However, voicing contrasts are not common in Australian languages, so testing this prediction is unlikely to yield meaningful results in this study area. Finally, Newman once again presents his findings in the form of a scale of “smallness”/“largeness” [16]. While consonants on the “large” end of Newman's spectrum are all voiced, voicing varies on the “small” end of the spectrum, with /p/, /n/, /d/, and /s/ at the extreme “small” end ([16], page 63). Newman's theory does not make predictions regarding natural classes. Expectations regarding the association of classes of consonants with magnitude meanings are little discussed in the literature.

### Hypotheses

If accoustic frequency is associated with magnitude symbolism in Australian languages, we expect to find more high-frequency sounds in sets of words with low-magnitude meanings than in the general vocabularies of the same languages, and more low-frequency sounds in words with high-magnitude meanings.

Based on the predictions above, our primary hypothesis is that vowels with high F2 will be associated with “small” or “proximal”) meanings. We also test for overall patterns associated with high and low vowels, as several existing studies predict some involvement of vowel height in magnitude symbolism. Predictions for consonant symbolism based on the existing literature are inconsistent and vague. We hypothesize that palatal consonants will be more likely to occur in “small”/“proximal” words, as these consonants produce a moderate amount of resonance at high acoustic frequencies and are pronounced farther forward in the oral cavity than velar consonants. They are also identified in Ultan, Nichols, and Jespersen as associated with diminutive meanings. We hypothesize that velar consonants will be more likely to occur in “large”/“distant” words, as they do not produce high-energy resonance at high acoustic frequencies and they are produced farther back in the oral cavity, relative to other sounds typical of Australian phoneme inventories. While velar consonants do not feature prominently in existing literature, they provide a contrast with the palatal consonants of the high-frequency category and are expected to involve less intense acoustic energy in the high frequencies. The two categories of acoustic frequency are thus each populated by a natural class of vowels and a set of dorsal consonants that contrast in just the type of acoustic parameters that have been previously associated with sound symbolsim. We also test for general associations between magnitude meanings and labial or lateral consonants. We do not expect to find sound symbolic associations with these classes of sounds, but we acknowledge that multiple categories of consonants could fit the vague “high acoustic freqency” criterion of the “frequency code” or the fronting pattern discovered by Ultan [7]. As noted above, we might also expect to find associations between consonant modal voicing and magnitude symbolism. However, few Australian languages make this distinction phonemically.

#### Size

Within individual languages, we expect to find that words with semantics related to ‘smallness’ will generally exhibit greater occurrence of front vowels than the overall phoneme frequencies of the sample languages would predict. Words with semantics related to ‘bigness’ are expected to be associated with a higher occurrence of back vowels. Overall, we expect to find a significantly higher occurrence of front vowels [i] and [e] in ‘small’ words and [o], [**ɔ**], and [u] in ‘big’ words than in general vocabulary for the same languages. We also expect to find more palatal consonants in ‘small’ words and velar consonants in ‘big’ words than in the general vocabulary.

#### Distance

We expect to find the same pattern of vowels as is hypothesized for size words, with ‘small’ words aligning with ‘proximal’ words and ‘big’ words aligning with ‘distant’ words. We also test for the prevalence of palatal consonants in proximal forms and velar consonants in distal forms, although these specific patterns have not been attested in previous studies.

## Materials and Methods

### The Australian Data

The 120 languages used in this study are all currently or formerly spoken on the Australian continent. One family, Pama-Nyungan, covers 90% of the area of the continent and comprises roughly two-thirds of the language total. The remaining languages are distributed among a further 27 families in the far north of the country [36]. To our knowledge, apart from one exception [38], there is no previous work on corporeal, synthetic, or conventional sound symbolism in Australian languages, and little work on imitative sound symbolism. Research in this last area focuses exclusively on ideophonic verbal constructions. Alpher [37], for example, describes features of ideophones in the Paman language Yir-Yoront. McGregor ([39], pages 324–333) and Schultze-Berndt [40] discuss preverbs in languages across northern Australia and identify a number of properties that some preverbs have in common with ideophones. Preverbs in languages such as Jaminjung are shorter than other words and contain a disproportionate number of final consonants and consonant clusters, for example.

Other work in Australian languages [41], [42] has noted the presence of onomatopoeia in several semantic domains, including bird names and acculturation terms. For example, in over 100 Australian languages the word for ‘crow’ (*Corvus orru*) contains the syllable *wak*. Examples of acculturation vocabulary include Nyangmuarta *ti**ŋ**kiti**ŋ**ki* ‘bell’, *minyawu* ‘cat’ and Bardi *bany* ‘bang (sound of a gun)’). However, while onomatopoeia has been identified in individual languages, there has been no systematic study of either this or sound symbolism in Australian languages more generally.

McGregor's [38] study of Gooniyandi sound symbolism is, to our knowledge, the only detailed study of sound symbolism in an Australian language. McGregor notes both onomatopoeia and conventional sound symbolism, and in particular ([39], page 328) the association with lamino-palatal consonants and small size words; compare *jiginya* ‘small’ with *nyamani* or *yagoowoo* ‘big’.

**Figure 1:**
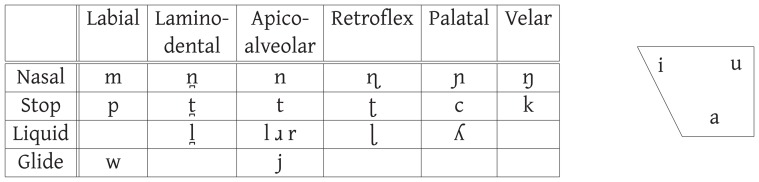
Common Australian Phoneme Inventory.

Australian languages are a good test case for the universality of sound symbolism. Previous work on magnitude and distance sound symbolism across the lexicon [7], [8], [30], which has identified associations between high vowels/palatals and “small”/“near” objects, has featured languages with moderate to large vowel inventories. Australian languages, however, typically have smaller vowel inventories [36], [43], [44]. Two-thirds of the languages in our sample, for example, have only three place distinctions (/i/, /a/, /u/) though they may also exhibit length contrasts at one or more places. In contrast, they have rich inventories of consonants with typically five or six places of articulation. The typical phoneme inventory is given in Figure 1.

**Figure 2:**
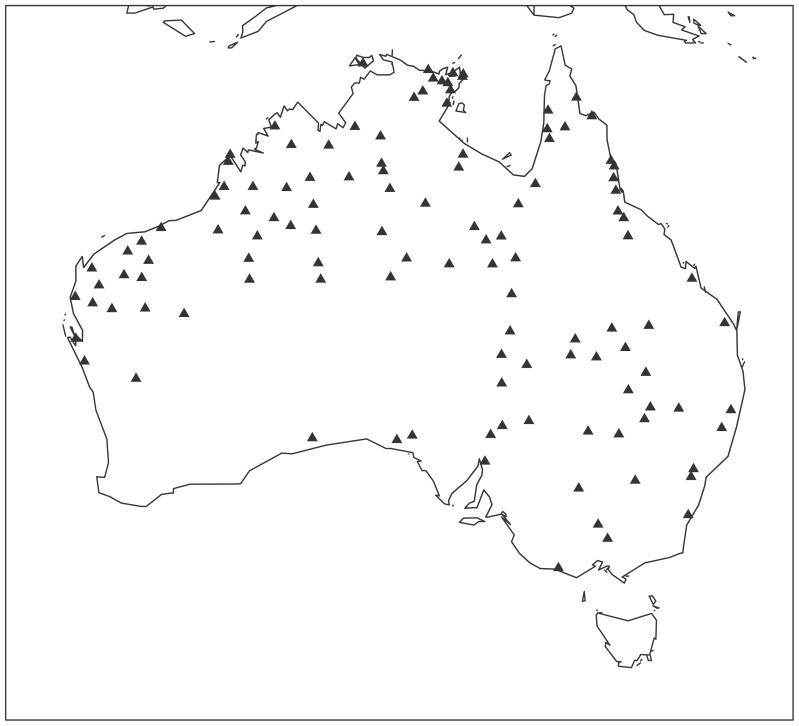
Languages in the Sample.

Data for this project was drawn from Bowern's comparative Australian lexical database (see Bowern [34] for more information). The languages included are given in Figure 2 below. They represent data from all Australian languages with more than 400 lexical items in the database, where the source information was in a phonemic orthography. Some sources in the database are extensive, but are written in orthographies which are non-standard and cannot be automatically converted to the standard orthography used in the database. Nekes and Worms [41], for example, has copious information about the languages of the Kimberley region but as Bowern [33] shows, the data are not consistently transcribed.

104 languages are members of the Pama-Nyungan family while the remaining 16 come from the Nyulnyulan, Worrorran, Bunuban, Gunwinyguan, Garrwan, and Maningrida Non-Pama-Nyungan families. The materials undersample Non-Pama-Nyungan regions because consistent lexical data were lacking for many families. Some Pama-Nyungan subgroups are also under-represented. The languages of the Southwest and Southeast (such as the Lower Murray languages; Horgen [45]) did not have sufficient phonemically transcribed materials to include. The languages of central Queensland are too poorly attested in lexicon to have sufficient wordlists, while many languages of the Paman subgroup, while well attested, are documented only in manuscript fieldnotes and are yet to be entered into the database. While we considered using data from non-phonemically transcribed sources, doing so would make accurate comparisons impossible. Because the non-phonemically transcribed materials make use of a range of English orthographic conventions in representing language sounds, it is impossible to reliably associate graphemes with phonemes. For example, the palatal stop /ty/ could be represented by <c, ch, tsh, sh, dy, dj, j, ty> or <tj>, depending on the context and source; some of these same graphemes are used to represent velar consonants, which would confound statistical frequency tests. Our current sample includes approximately 30% of the languages of Australia, which is sufficient to draw conclusions about the languages. Figure S1 gives the languages and forms used for the sound symbolism data set.

We approach the investigation of sound symbolism in two ways. In the first case, we ask whether Australian languages *overall* provide evidence for magnitude sound symbolic patterning. We pool words from all languages in the sample to test for the categories of phonemes which appear with greater than expected frequency in magnitude related vocabulary, as compared to the rest of the lexicon. Since the number of words in each language is small, it is possible that phoneme frequencies in each category might be significant due to skewing, rather than being reflective of sound symbolic marking. Pooling data from languages minimizes this risk. However, we also investigate patterns within individual languages, to gauge the extent of support for sound symbolic marking across the sample. Because some languages are better attested than others, combining data from multiple languages may give misleading results, if only the best attested languages exhibit sound symbolic tendencies. However, as we see below, this is not the case, and sound symbolic marking is attested across the continent.

### Dataset

Size and distance vocabulary words were compiled from the lexica by tagging all translations of the words given in Table 1. The words were divided into semantic fields and tagged for expectations of whether they should cluster in the “high/small” frame or “low/large” frame based on their meaning. This yielded a total of 6,656 items across the 120 languages.

**Table 1:** Size and Distance Vocabulary.

Lexical Item	Category	Condition	Lexical Item	Category	Condition
to	distance	high	small	size	high
low	distance	high	little	size	high
shallow	distance	high	skinny	size	high
towards	distance	high	short	size	high
narrow	distance	high	thin	size	high
here	distance	high	wide	size	low
these	distance	high	fat	size	low
closeby	distance	high	large	size	low
near	distance	high	big	size	low
this	distance	high	tall	size	low
there	distance	low	long	size	low
that	distance	low			
those	distance	low			
that over there	distance	low			
those over there	distance	low			
that yonder	distance	low			
those yonder	distance	low			
over there	distance	low			
yonder	distance	low			
far	distance	low			
deep	distance	low			
away	distance	low			
high	distance	low			
from	distance	low			

Size and distance vocabulary words were compiled from the lexica by tagging all translations of the following words. The words were divided into semantic fields and tagged for expectations of whether they should cluster in the “high/small” frame or “low/large” frame.

General vocabulary lists consist of the entire lexical sample available for each language. The database from which these general lexical samples were drawn is populated primarily by items of basic vocabulary, flora and fauna terminology, material culture terms. Though the general vocabulary lists were not edited to control for length, part of speech, or other characteristics of the forms they contain, our inclusion of only those languages with at least 400 items in this general list should provide sufficient data to provide a basic sense of the overall frequencies of various classes of phones in these languages. Although some items in the list may participate in some forms of sound symbolism (e.g. bird names may involve onomatopoeia in the form of reduplication), the few items in the list that could potentially be associated with phonetic iconicity are unlikely to have a large effect on the overall phone frequencies of the large samples. Limited knowledge of sound symbolic patterns and processes, especially in less-studied languages, prevents us from constructing a general vocabulary list that excludes such items.

Phonemes in the lexical dataset were categorized by natural classes. Classes representing front vowels, back vowels, palatal consonants, and velar consonants were selected for analysis because these classes correspond to existing claims regarding acoustic frequency (in particular vowel F2 and palatal consonant frequency) and magnitude symbolism. Three front vowels and five palatal consonants constitute the HIGH class of sounds; four back vowels and four velar consonants constitute the LOW class. This categorization scheme creates two groups of sounds expected to correspond to opposite types of meanings, each containing both consonants and vowels and representing roughly equal numbers of phonemes.

### Methods

Cross-linguistic studies of size- and distance-based sound symbolism typically identify pairs or groups of words with proximal/distal or large/small meaning contrasts and quantify patterns based on the number of languages that exhibit specific sounds in these words [7], . This study, however, compares the occurrence of sounds hypothesized to be associated with magnitude-related meanings in words with those meanings versus in the total available lexical sample. This approach avoids the conundrum noted by Bauer ([14], page 192): if a particular sound that occurs in a smallness-denoting item is a very frequent sound in a particular language, does its occurrence in that word indicate symbolism? By comparing the relative frequencies of sounds in symbolism-associated lexical subsets with the overall frequencies of these sounds, we gain a measure of whether the occurrence of sounds in these subsets is *significantly* higher than what we might expect, given the overall frequencies of phonemes in the language.

Using the lexical dataset described above, we apply a paired t-test to data describing the percentage of words in the symbolism-associated meaning category that contain a particular sound or set of sounds and the percentage of words containing that sound in the general lexical sample, for a single set of languages. We compare the percent occurrence of expected symbolic sounds out of all of the phonemes in the sample using the same method. For each test, we include only those languages for which a particular sound is possible (e.g. if a particular language does not include the phoneme /dy/ in its inventory, it will be excluded from the t-test of /dy/ occurrence in smallness-denoting versus general lexical items). These tests yield p-values which are used to assess whether the overall occurrence of a particular sound in the size/distance lexical datasets is significantly different than its overall occurrence in the sample languages.

For each type sound-meaning correspondence investigated, we report two t-test results. The first compares the number of magnitude-expressing words containing the relevant class of sounds to the total number of words in the general lexical sample that contain those same sounds. The second emphasizes counts of individual sounds, comparing the number of occurrences of particular phonemes in the magnitude-related lexical set with the number of occurrences of those same phonemes in the general lexical dataset. This provides an indicator of whether more words in the relevant meaning category contain the symbolic sounds than we would expect, given the general distribution of those sounds, as well as an indicator of whether the symbolic category of sounds makes up a greater portion of the overall pool of sounds used in the relevant meaning category than in the same languages' general vocabularies. Existing work on sound symbolism has not included specific claims regarding the way that sound symbolism might be instantiated, so we include both logical measures.

Finally, we also examined the effect of positional prominence, by comparing overall occurrence of a sound or sound class with its occurrence in initial position. Previous work in linguistics [46], [47] has identified the initial position in a word as ‘prominent’ or ‘marked’. For example, languages often make all phonemic distinctions in initial position, whereas some of these distinctions might be collapsed in other positions. McGregor [38] found a positional effect in Gooniyandi sound symbolism, though the effect was for final, rather than initial, position. We might therefore expect to see a positional effect across the sample.

Unlike studies which identify sound symbolism on a lexical item-by-lexical item basis, this methodology yields summary statistics that are useful for understanding patterns of sound symbolism across the entire language sample. However, it does not identify individual languages that show evidence of magnitude-based sound symbolism. To better understand the distribution of size/distance sound symbolism in Australia, we test the occurrence of expected symbolic sounds in size/distance and general vocabulary sets using Fisher's exact test. The p-values associated with this test can be used to identify which individual languages exhibit evidence of particular sound-meaning correspondence.

Finally, we test for areal patterns of sound symbolism within the dataset by mapping the residuals of a correlation between the occurrence of a sound in the size/distance category and its ocurrence in general lexical data. Moran's I is used to test for patterns of spatial autocorrelation in the resulting maps. We discuss these results and compare them to genealogical classifications.

## Results

The results of this procedure demonstrate significant associations between meanings of “smallness” and “nearness” and the expected front vowels and palatal consonants, with slightly weaker patterns linking “large” and “distant” meanings to back vowels and velar consonants. Contrary to expectations, the most unambiguous patterns identified in this sample link palatal consonants to “small/near” meanings and velar consonants to “large/far” meanings, while the associations between vowels and symbolism are obscured in certain cases by variation associated with positional prominence and sampling effects associated with phonotactic constraints.

### Overall HIGH Condition

Considering all smallness- and nearness-denoting items as a single conceptual category expected to be represented by high-frequency sounds, we find considerable supporting evidence for symbolism in these languages. As reported in Table 2, the total occurrence of high-frequency sounds as a percentage of all phonemes is higher in this semantic class than in general vocabulary. Palatal consonants occur in a significantly higher percentage of words in this semantic category and comprise a greater percentage of total phonemes than in the general vocabulary sample. Contrary to our expectations, however, front vowels do not occur significantly more frequently in “small/near” vocabulary than in general vocabulary. This is perhaps unexpected, given Ultan's [7] findings that vowel fronting is the most robustly attested form of magnitude symbolism and that consonant symbolism patterns are more variable.

**Table 2:** Mean percent of words containing high-frequency sounds and mean overall percent occurrence of high-frequency sounds.

Condition	Basic Vocabulary	“High” Vocabulary	p
Words containing palatal	27.20%	30.99%	0.0072**
Words containing V front	46.74%	45.85%	0.6854
Total palatal sounds	5.33%	6.30%	0.0022**
Total V front	11.11%	11.52%	0.2328
Total HIGH sounds	15.54%	16.79%	0.0190*

When broken down by position in the word, as in Table 3, both palatals and front vowels are significantly more frequent in initial and final positions in the “small/near” category than in the general vocabulary set. Palatal consonants are further shown to be significantly more common in medial positions in “small/near” words than in general vocabulary, but front vowels do not follow this pattern in medial positions. The sheer number of medial vowels in the dataset, combined with this position's low phonological prominence, likely explains the unexpectedly low occurrence of front vowels in medial positions of “small/near” words. More generally, however, the patterns reported in Table 3 seem to demonstrate robust support for the frequency-magnitude hypothesis.

**Table 3:** Mean percent of lexical items containing high-frequency sounds, by position in word.

Condition	Basic Vocabulary	“High” Vocabulary	p
Intial HIGH segment	12.28%	22.21%	4.78E-09**
Initial palatal	12.16%	20.51%	5.00E-07**
Initial V front	6.71%	19.63%	0.0014**
Final HIGH segment	22.09%	29.25%	1.07E-05**
Final palatal	6.72%	11.63%	0.0031**
Final V front	20.94%	26.88%	0.0002**
Medial palatal	16.87%	20.81%	0.0022**
Medial V front	34.90%	33.99%	0.7034

### Overall LOW Condition

The overall low frequency/high magnitude patterns shown in Table 4 are strikingly less supportive of the overall frequency-magnitude hypothesis than their high-frequency counterparts in Table 2. Only velar sounds are significantly more common in the “large/far” vocabulary, and this pattern barely falls below the p<0.05 threshold for significance. This is a surprising result in light of the focus on vowel quality in the magnitude-symbolism literature and the silence of earlier researchers regarding velar sounds and “large/far” meanings. The general finding that low-frequency symbolism is not as robustly supported as high-frequency symbolism in this dataset suggests that this frequency-magnitude mapping may not be implemented evenly at both ends of the spectrum. In light of Thompson and Estes' [17] assertion that size sound symbolism is gradient in nature, this is a surprising finding. Whereas Thompson and Estes [17] found clear associations between sizes of objects and the F2 of sounds used to name them at both ends of the size spectrum as well as in the middle, our results show a stronger relationship at the small-magnitude/high-frequency end of the scale. This one-sided correlation is more consistent with a contrastive view of sound symbolic features, or even a system where high and low-frequency symbolism may be employed independently by languages, not necessarily in opposition to one another.

**Table 4:** Mean percent of words containing low-frequency sounds and mean overall percent occurrence of low-frequency sounds.

Condition	Basic Vocabulary	“Low” Vocabulary	p
Words containing velar	50.18%	51.85%	0.1944
Words containing V back/round	47.59%	45.26%	0.9232
Total velar sounds	10.87%	11.69%	0.0445*
Total V back/round	11.37%	11.01%	0.8079
Total LOW sounds	21.48%	21.93%	0.2673

Dissecting the overall low-frequency results by position in the word, as in Table 5, the evidence for low-frequency/high-magnitude iconicity appears much stronger. In the prominent word-inintial position and in word-final position we find significantly more low frequency sounds in “large/far” words than in general vocabulary. The medial positions, which are presumably of lower salience in words in this sample, show no difference between “large/far” meanings and general vocabulary, with regard to low-frequency sounds. Due to the imbalance in the number of sounds that occur in each of these positions (i.e. the fact that each word has a single initial and final segment, but may have many medial segments), this undifferentiated distribution in medial positions contributes heavily to the lack of significant findings in Table 4.

**Table 5:** Mean percent of lexical items containing low-frequency sounds, by position in word.

Condition	Basic Vocabulary	“Low” Vocabulary	p
Initial LOW segment	25.85%	30.55%	0.0051**
Initial velar	26.02%	30.06%	0.0141*
Initial V back/round	6.14%	13.95%	0.0069**
Final LOW segment	18.35%	23.16%	6.00E-05**
Final velar	12.16%	16.69%	0.0210*
Final V back/round	16.97%	21.10%	0.0005**
Medial velar	29.75%	31.65%	0.1436
Medial V back/round	47.93%	50.19%	0.2176

### Size-related Conditions

#### Small

Focusing specifically on size, we find very similar results to the overall patterns found for high- and low-frequency magnitude symbolism (see Table 6). As with the overall “small/near” category (that is, the HIGH condition discussed above), the size subset of expected high-frequency iconic forms shows a significant overall association between high-frequency sounds and “small” vocabulary, and significant links between palatals and “small” meanings. However, as with the overall condition, we do not find a significantly greater occurrence of front vowels in “small” words than in general vocabulary. In fact, the percentage of words containing front vowels and the percentage of front vowels in the entire set of phonemes are higher for the general vocabulary sample than the “small” vocabulary. This is in contrast with Ultan's ([7], page 554) finding that “front vowels predominantly correspond to diminutive and associated categories”.

**Table 6:** Mean percent of words containing high-frequency sounds and mean overall percent occurrence of high-frequency sounds.

Condition	Basic Vocabulary	“Small” Vocabulary	p
Words containing palatal	26.35%	40.15%	8.21E-08**
Words containing V front	47.72%	46.75%	0.6647
Total palatal sounds	5.09%	7.87%	3.29E-07**
Total V front	11.37%	10.84%	0.7958
Total HIGH sounds	14.15%	15.85%	0.0072**

When broken down by position in the word (as shown in Table 7), we find that high-frequency sounds are, in general, significantly associated with “small” meanings in initial and final positions, much as we found for overall high-frequency symbolism in Table 3. A notable exception to this pattern is the non-significant difference between the occurrence of initial front vowels in “small” vocabulary and general vocabulary, in spite of the relatively large differences in the means of these two categories. The rarity of languages in the sample which allow initial vowels and the principle of excluding languages for which no relevant data exists (rather than including artifactual zero data) lead to a sample of only 2 languages in which to test initial front vowels across “small” and general vocabulary. While this result technically contradicts our hypothesis, the p value for this particular test is highly sensitive to the distribution of individual data points and is unlikely to be a reliable indicator of significance for this particularly small sample. In sum, we find good evidence here for the association of high-frequency sounds with “small” meanings in initial and final positions, as we found for the overall “small/near” category, and we further find a significantly higher ocurrence of palatal sounds in medial positions in “small” vocabulary items than in similar positions in general vocabulary.

**Table 7:** Mean percent of lexical items containing high-frequency sounds, by position in word.

Condition	Basic Vocabulary	“Small” Vocabulary	p
Intial HIGH segment	9.98%	31.52%	2.48E-24**
Initial palatal	9.82%	30.99%	1.45E-24**
Initial V front	7.51%	25.56%	0.1908
Final HIGH segment	22.66%	31.31%	6.76E-05**
Final palatal	7.09%	15.88%	0.0038**
Final V front	22.39%	29.80%	0.0008**
Medial palatal	16.88%	32.12%	4.98E-08**
Medial V front	36.06%	38.59%	0.1293

#### Large

For vocabulary with “large” size meanings, we find a significant association with back vowels in terms of the number of words containing back vowels, but not for the overall percentage of phonemes that are back vowels. There is no significant overall tendency for velar consonants to occur more frequently in “large” vocabulary than general vocabulary. This contrasts with the overall low-frequency symbolism, for which velar sounds showed the most significant association with magnitude.

Decomposing the sample by the position of sounds in the word, we find stronger evidence for sound symbolic patterns (see Tables 8 and 9). In every position, low sounds are significantly more frequent in words with “large” meanings than their overall distribution in the same languages would predict. The seeming contrast between these patterns and the general trends in Table 8 is accounted for by words that contain low-frequency sounds in multiple positions in addition to the variance in individual samples.

**Table 8:** Mean percent of words containing low-frequency sounds and mean overall percent occurrence of low-frequency sounds.

Condition	Basic Vocabulary	“Large” Vocabulary	p
Words containing velar	50.04%	53.45%	0.0757
Words containing V back	48.43%	54.16%	0.0149*
Total velar sounds	10.85%	11.61%	0.0899
Total V back/round	11.60%	11.99%	0.3019
Total LOW sounds	19.50%	20.52%	0.1458

**Table 9:** Mean percent of lexical items containing low-frequency sounds, by position in word.

Condition	Basic Vocabulary	“Large” Vocabulary	p
Initial LOW segment	23.81%	34.45%	0.0007**
Initial velar	26.02%	34.33%	0.0085**
Initial V back/round	4.64%	30.44%	0.0020**
Final LOW segment	18.70%	32.22%	1.23E-06**
Final velar	13.96%	29.42%	0.0009**
Final V back/round	18.62%	30.33%	0.0001**
Medial velar	29.77%	43.51%	1.36E-07**
Medial V back/round	41.08%	48.73%	0.0026**

### Distance-related Conditions

#### Near

By overall measures, “near” words appear to be associated with all of the expected markers of sound symbolism. This would seem to confirm Ultan's ([7], page 546) conclusion that there is better cross-linguistic support for distance symbolism than size symbolism. To date no satisfactory explanation for this trend has been put forth. It is possible that the coding of contrasts in grammatical morphemes like demonstratives and deictic expressions leads to the implementation of phonemic contrasts to signify distance that may not be as widely exploited in lexical expressions denoting smallness. If such an explanation were plausible, we would also expect to find a difference in the prevalence of symbolism in grammatical expressions of size (e.g. diminutive and augmentative morphemes) and lexical morphemes expressing size (e.g. adjectival or nominal roots). Australian languages tend not to exhibit inflectional marking for diminution or augmentation so this hypothesis cannot be tested with the available materials; we note, however, that both Ultan [7] and Nichols [30] made heavy use of inflection marking of size in studying sound symbolic tendencies. Further broad cross-linguistic study would be required to arrive at a better understanding of the relatively stronger support for distance symbolism than size symbolism.

Not surprisingly, given the significance of the iconic associations in Tables 10 and 11, sounds expected to be associated with “near” meanings are significantly more common in every position. Particularly striking are the differences between “near” and basic vocabulary in the occurrence of high frequency sounds in the prominent initial position.

**Table 10:** Mean percent of words containing high-frequency sounds and mean overall percent occurrence of high-frequency sounds.

Condition	Basic Vocabulary	“Near” Vocabulary	p
Words containing palatal	30.62%	36.69%	0.0057**
Words containing V front	47.26%	58.04%	8.52E-05**
Total palatal sounds	5.99%	7.71%	0.0004**
Total V front	11.26%	16.09%	3.71E-07**
Total HIGH sounds	14.82%	20.63%	2.22E-08**

**Table 11:** Mean percent of lexical items containing high-frequency sounds, by position in word.

Condition	Basic Vocabulary	“Near” Vocabulary	p
Intial HIGH segment	9.98%	31.52%	6.47E-09**
Initial palatal	14.36%	29.90%	1.47E-06**
Initial V front	6.44%	41.22%	0.0003**
Final HIGH segment	22.66%	31.31%	2.54E-09**
Final palatal	7.09%	15.46%	0.0090**
Final V front	20.66%	37.66%	2.16E-08**
Medial palatal	19.17%	28.49%	0.0004**
Medial V front	35.33%	44.52%	0.0012**

#### Far

The robust support for distance symbolism extends into the low-frequency categories. Tables 12 and 13 show a significant link between the overall occurrence of low-frequency sounds and “far” meanings as well as significant associations with “distance” within the sub-categories of velar consonants and back vowels. The percentage of words containing back vowels is not significantly higher for “far” meanings than for general vocabulary, however. This pattern is influenced, to some extent, by the general frequency of back vowels in the language – these sounds occur in roughly half of the words, on average, in the Australian language samples.

**Table 12:** Mean percent of words containing low-frequency sounds and mean overall percent occurrence of low-frequency sounds.

Condition	Basic Vocabulary	“Far” Vocabulary	p
Words containing velar	49.76%	57.40%	0.0028**
Words containing V back	47.93%	50.19%	0.2176
Total velar sounds	10.77%	13.52%	7.77E-05**
Total V back	11.38%	13.49%	0.0103*
Total LOW sounds	19.84%	24.21%	0.0002**

**Table 13:** Mean percent of lexical items containing low-frequency sounds, by position in word.

Condition	Basic Vocabulary	“Far” Vocabulary	p
Initial LOW segment	25.88%	45.68%	4.57E-08**
Initial velar	26.18%	44.33%	2.43E-07**
Initial V back/round	7.28%	48.81%	0.0636
Final LOW segment	17.04%	31.13%	5.07E-09**
Final velar	11.04%	20.53%	0.0312*
Final V back/round	16.76%	30.55%	4.38E-08**
Medial velar	29.73%	33.50%	0.0766
Medial V back/round	40.49%	43.44%	0.1517

As with “small” vocabulary items, the small number of languages that allow initial back vowels at all results in a very small sample size for the t-test comparing the occurrence of back vowels in “far” vocabulary and general vocabulary. For this particular test, N = 4, making it the second-smallest sample in the study. In this condition, statistical significance is difficult to test, and the p value over the 0.05 threshold for initial back vowels is less secure than the other results. The large difference in means can be interpreted somewhat cautiously as a weak, nonstatistical form of support for the frequency hypothesis. Overall, then, the results by position echo the overall finding that low-frequency sounds occur more frequently with high-magnitude meanings than would be predicted by their distributions in general vocabulary data.

### Individual languages

The use of simple t-tests to assess differences in phoneme distributions between magnitude-related and general lexical samples allows the effect of sound symbolic processes to be distinguished from general distributional trends for various sounds. We find that the iconic representation of magnitude by certain categories of sounds occurs in Australian languages when considered as a group, and that the associations predicted by the frequency-magnitude hypothesis are generally substantiated in our sample. Symbolic sound patterns involving word-initial and word-final segments are more robustly supported than those involving medial segments, however, and counter to our expectations, patterns involving consonants are more securely attested than those involving vowels.

In addition to considering the patterns of sound symbolism across the continent, we also investigated pattern significance in individual languages. Here we found considerable variation, both in the languages which show significant patterns and the phonemes used to signal sound symbolic categories. However, as might be expected, the data overall reflects the same patterns described above. 65 (or 54%) of the languages in the sample showed significant results for one or more of the categories. This is markedly higher than the 28% participation in magnitude sound symbolism reported by Ultan. The most consistent languages for sound symbolism marking were Ngarluma, Djabugay, Paakintyi, Martu Wangka, and Pintupi-Luritja, with 8 (Ngarluma), 6 (Djabugay), and 5 (Paakintyi, Martu Wangka, Pintupi-Luritja) categories marked. Compare Djabugay *pa**ŋ**kal* ‘big’ versus *pipuy* ‘small’, *wakarra* ‘wide’ versus *wiki* ‘narrow’, *kalkalay* ‘tall’ versus *wanti* ‘short’, and *kakay* ‘far’ versus *pirri* ‘near’. Twenty-five languages marked a single category.

In the HIGH condition, front vowels were significant markers in 17 languages from across the continent, and palatals were significant for 12 languages. The only language to have significant results for both categories was Ngarluma. Ngarluma also showed significant velar marking in the overall LOW condition. 14 languages reached significance here; 9 languages had significant results for velar marking in the LOW condition. The Wati languages Martu Wangka [31] and Pintupi-Luritja [32] showed significant results for both back vowels and velars in the overall LOW condition, as did the Nyulnyulan language Bardi [33].

Within individual categories, the LOW distance condition was most widely marked, with 15 languages providing significant results. There are six languages which show support for both velar and back vowel marking in this condition. Some languages show significance for the HIGH condition in one category but the LOW condition in the other. For example, the Yol**ŋ**u language Dhay'yi significantly marks the HIGH condition for distance, but the LOW condition for size. Compare *galki* ‘close’ and *bathala* ‘big’, for example.

Australian languages thus differ in the extent to which they make use of sound symbolic tendencies. However, when they *do* make a distinction, the same phonemic categories tend to be lexicalized in the same way. This provides further support for the cross-linguistic validity of sound symbolic categories.

### Additional patterns

In addition to the sound categories associated with our primary hypotheses, we tested for overall associations between high vowels, low vowels, labial consonants, and lateral consonants with high-magnitude or low-magnitude meanings. Results of these t-tests are reported in Table 14. We found an association between low vowels and LOW condition meanings which is highly significant for both percentage of words containing low vowels and percentage of all phonemes that are low vowels. We also found that high vowels make up a significantly higher percentage of the phonemes in HIGH condition words, although the overall percentage of HIGH-meaning words containing high vowels was not significantly different than general vocabulary. We found absolutely no evidence for associations between lateral consonants or labial consonants and magnitude-related vocabulary.

**Table 14:** T-test results for additional phoneme classes.

	HIGH - words	LOW - words	HIGH - phones	LOW - phones
Laterals	1.0000	1.0000	1.0000	1.0000
Labials	1.0000	1.0000	1.0000	1.0000
High vowels	0.7486	0.9679	0.0233*	0.9874
Low vowels	0.9838	0.0008**	0.8914	3.18E-07**

## Discussion

### Contrary Correlations

To accurately interpret the results reported in the previous section, we must also consider the possible evidence for sound-meaning links that contradict the initial hypotheses. A comparison of the basic patterns associated with our hypotheses (Table 15) and the opposite associations between frequency and magnitude (Table 16) reveals several unexpected insights. First, we find a significant overall association between low-frequency sounds (i.e. velars and back vowels) and low-magnitude lexical items (i.e. “small” and “near” meanings). The association between low frequency sounds and low magnitude items, listed in Table 16, appears to be a direct contradiction of the frequency-magnitude hypothesis. However, it is important to note that low-magnitude lexical items are also significantly associated with high-frequency sounds in these languages. In other words, “small” and “near” meanings are associated with the sounds included in both our high-frequency category and our low-frequency category. This result need not be interpreted as contradictory. Rather, it suggests that the phonetic characteristics used to classify sounds as high-frequency or low-frequency do not fully capture the relevant generalizations for magnitude symbolism in Australian languages. Further examination of the data reveals that this unexpected finding for “small” and “near” meanings is related to a high occurrence of high back vowels with these low-magnitude meanings (p = 0.0058*), whereas velar consonants are not significantly associated with these meanings (p = 0.7179). This point reinforces the findings of Diffloth [21], that there can be language-specific significant sound symbolic categories.

**Table 15:** Mean percent occurrence of iconic sounds in magnitude-related and general vocabulary.

Condition	Basic Vocabulary	“Magnitude” Vocabulary	p
Overall HIGH/Low magnitude	15.54%	16.79%	0.0190*
Overall LOW/High magnitude	21.48%	21.93%	0.2673
HIGH/Small	14.15%	15.85%	0.0072**
LOW/Large	19.50%	20.52%	0.1458
HIGH/Proximal	14.82%	20.63%	2.22E-08*
LOW/Distant	19.84%	24.21%	0.0002**

**Table 16:** Mean percent occurrence of unexpected iconic sounds in magnitude-related and general vocabulary.

Condition	Basic Vocabulary	“Magnitude” Vocabulary	p
Overall HIGH/High magnitude	15.98%	13.98%	0.9960
Overall LOW/Low magnitude	21.95%	23.69%	0.0349*
HIGH/Large	15.99%	16.39%	0.3598
LOW/Small	21.94%	26.69%	0.0319*
HIGH/Distant	16.08%	13.08%	0.9979
LOW/Proximal	21.83%	21.42%	0.6468

Splitting the overall low magnitude category into size and distance subsets, we find that the same counterintuitive association with low frequency sounds occurs for “small” vocabulary but not for “proximal” meanings. Further splitting the low frequency category into the consonant and vowel classes that it is comprised of reveals that the ultimate source of these significant, counterintuitive findings is a relationship between “small” meanings and back vowels (p = 1.87E-06*). Velar consonants, as the frequency-magnitude hypothesis would predict, are not significantly associated with “small” meanings (p = 0.5865). Although the expected association between “small” vocabulary and high frequency sounds was found to be significant only in word-final position, it is not necessarily the case that “small” meanings are associated with back vowels instead of the expected front fowels.

It is reasonable to interpret this apparent conflict in mapping frequency to meaning as evidence that the front/back vowel contrast is not the relevant parameter for “smallness” sound symbolism in Australian languages. The low vowel /a/, interpreted as a central vowel for the purposes of this study, is not included in either the front or back vowel categories. Hence, it is likely that the relevant contrast for symbolizing “smallness” is vowel height (i.e. F1 in the acoustic signal), rather than vowel backness (i.e. F2). Though this is a contradiction to formulations of the frequency-magnitude hypothesis that assess vowel frequency using F2, it is consistent with Ultan's [7] finding that vowel height contrasts are a relatively common correlate of magnitude symbolism.

### Spatial patterns

Cross-linguistic studies of sound symbolism frequently grapple with the question of whether observed patterns reflect universal communicative patterns, or whether the occurrence of patterns across languages stems instead from common genealogical inheritance or areal convergence (cf. for example [30]). Below we discuss the evidence for symbolism in individual languages and the evidence that exists for the common inheritance of sound symbolic patterns in closely related languages. While genealogical relationships between the Australian languages do not explain the general distribution of sound symbolic patterns in this continent, it is important to consider whether areal processes might. To do this, we map the residuals of a regression between the ocurrence of symbolic sounds in magnitude-related vocabulary and their occurrence in general vocabulary for each language. The residual variation, in this case, serves as a measure of the amount of variation in the distribution of these sounds that could be associated with sound symbolism. Mapping these figures gives us a rough representation of where we find more or less evidence for sound symbolism. We use Moran's I, a common measure of spatial autocorrelation, to test for spatial patterns in these residuals [35]. This metric compares values across spatial neighborhoods to determine whether there is significant evidence of clustering (indicated by a Moran's I value approaching 1) or even dispersal (indicated by a Moran's I value approaching −1). Moran's I values approaching zero indicate essentially random spatial distributions.

Moran's I values for each of the overall patterns found to be significant are shown in Table 17. The small, positive Moran's I values listed in Table 17 demonstrate that the spatial organization of languages that exhibit sound-meaning correspondences associated with symbolism is essentially random. If areal spread of symbolic sound-meaning associations were responsible for the occurrence of sound symbolism in Australia we would expect significant spatial clustering of the languages that show evidence of these associations. The absence of spatial patterns suggests that unlike sound symbolic patterns in the North American languages sampled by Ultan [7] and Nichols [30], areal spread is an unlikely explanation for the occurrence of sound symbolic processes in Australian languages.

**Table 17:** Moran's I for significant general frequency-magnitude patterns.

Condition	Moran's I
Overall HIGH	0.042
HIGH/Small	0.026
HIGH/Proximal	0.045
LOW/Distant	0.016

### Historical relatedness

**Figure 3:**
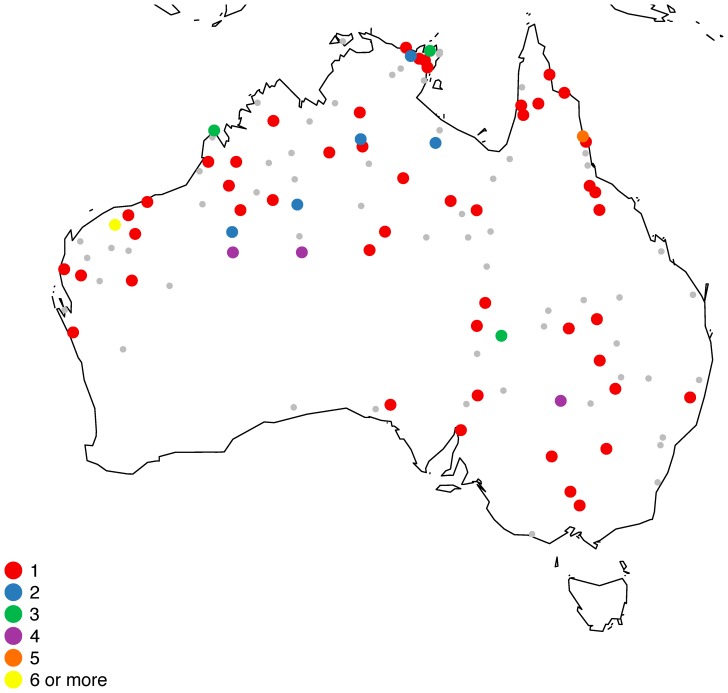
Languages in the Sample with Significant Sound Symbolism Marking. Small gray dots denote the locations of languages without significant magnitude sound symbolism marking.

The languages which show significant categories are given in Figure 3 below. They are found across both Pama-Nyungan and Non-Pama-Nyungan regions, and across the primary subgroups of Pama-Nyungan. The only apparent genealogical clusters are among the Wati (Western Desert) languages, where several Wati varieties show recurrent sound symbolic patterns, and among the Yol**ŋ**u languages of eastern Arnhem Land. Both subgroups are quite closeknit, with the languages exhibiting multiple lexical cognates in the relevant domains (such as *purlka* ‘big’ in Wati). In fact, it is perhaps surprising that we do not see more effects of this type, given the shared relationships among Pama-Nyungan languages. Some of the words in these domains, however, are subject to rapid lexical replacement. The translation equivalent for ‘big’, for example, has 175 distinct cognate sets across 289 Pama-Nyungan languages [34], [49], with 165 of those forms occurring in only one or two languages.

To further explore possible effects of historical relatedness as a factor in sound symbolism, we tested for phylogenetic signal of a sound symbolism trait. To do this, Blomberg's *K* [48] was estimated for the 104 Pama-Nyungan languages in the sample. This statistic allows us to infer whether the distribution of a trait across a tree shows greater than expected phylogenetic signal, given a model where characters evolve stochastically through time. We used the phylogenetic tree from Bowern and Atkinson [49] to map sound symbolism characters onto a phylogeny, and statistics were calculated with the R package Picante [50]. There is no consensus about the structure of the relationships between Pama-Nyungan and Non-Pama-Nyungan languages, and so our sample used only Pama-Nyungan languages. The traits used were whether the tip language marked sound symbolism for the ‘high’ or ‘low’ categories, or for size or distance symbolism. Blomberg's *K* varied between 0.32 and 0.36 for these categories, indicating no support for phylogenetic signal in the trait distribution. PIC variance was not significant for any of the characters, indicating that the traits are not distributed phylogenetically.

Given the surprisingly low occurrence of shared sound symbolic patterns within Pama-Nyungan subgroups, the scattered distribution of individual sound-meaning patterns, and the overall widespread occurrence of magnitude-related symbolism in both Pama-Nyungan and Non-Pama-Nyungan languages, we conclude that genealogical inheritance plays a surprisingly small role in accounting for the evidence of sound symbolism presented above.

### Conclusions

In all, the results presented above show support for the same types of sound-meaning correspondences that have been argued to exist in languages elswhere in the world. In particular, the link between consonants with a close/front articulation and “small”/“near” meanings that has been suggested by earlier studies is well-supported by our finding that palatal consonants are more often found in these words than in general vocabulary [7], [18]. Though Australian languages were not included in the language samples on which these existing hypotheses were based, the distributions of the relevant categories of sounds in Australian languages generally fit the predictions of these theories. In the details, however, we find several deviations from these general predictions. These nuances in the sound-meaning associations we find evidence for have some impact on the conclusions we can draw about the nature of magnitude sound symbolism.

Compared to Ultan [7], whose sound symbolism study included a relatively large language sample and examined a wide range of sound symbolic patterns, we find a greater involvement of palatal and velar consonants in the expression of “small”/“near” and “large”/“far” meanings respectively. Whereas Ultan found vowels to be involved in the dominant sound symbolism patterns in his sample, we find stronger statistical support for sound symbolism reliant on palatal and velar consonants than for vowel V2-related symbolism. However, the significant associations we find between high vowels and “small”/“proximal” meanings and low vowels and “large”/“distant” meanings indicate that vowel expression of magnitude symbolism is not limited to F2, and in fact height may play a significant role in mapping magnitude to sound in these languages. Phonological differences between the languages of Ultan's North America-skewed sample and Australian languages may help to explain the relatively weaker involvement of consonants in that study. However, our overall findings regarding the strength of the consonant patterns and the nature of the vowel patterns are still unanticipated. Our results suggest that in spite of general discussion of “high acoustic frequency” consonants participating in magnitude symbolism, existing literature has overlooked an important manner in which magnitude symbolism may be expressed. Although the palatal and velar classes of sounds we investigate have received little systematic study as sound symbolic categories, the significant associations reported here for these categories, and the absolute absence of such patterns for other classes of consonants tested in this study, provide solid evidence for the involvement of these classes of sounds in magnitude symbolism. The significant vowel height patterns we report also suggest that characterizations of magnitude-vowel symbolism that rely on F2 may be unduly simplifying the mapping between vowel quality and magnitude.

We might expect, in light of the discussion of magnitude symbolism in the literature [7], [15]–[17], [20], that links between high-magnitude meanings and low frequency sounds should be just as important as associations between high frequency sounds and low-magnitude meanings. However, the relationship between low-frequency sounds and “large”/“far” meanings is weaker than than the association of high-frequency sounds with “small”/“near” meanings in our sample of Australian languages. This suggests that magnitude symbolism, at least in some languages, may be better thought of as a mapping between classes of sounds and meanings, rather than a system that necessarily employs a contrastive distinction in acoustic properties to encode a contrast in meaning.

The frequency hypothesis put forth by Ohala [18], which explains the expected patterns in magnitude symbolism through an appeal to general functions of acoustic frequency in animal communication, is similarly consistent with our most general findings for Australian languages. However, the asymmetry between the iconic patterns associated high and low frequency, noted above, weakens the support that overall sound-meaning patterns in Australian languages provide for the frequency hypothesis. On the other hand, our finding that palatal consonants occur more frequently in “small”/“near” words while velar consonants occur more frequently in “large”/“far” words is, indeed, consistent with the Ohala [18] frequency hypothesis. Further testing of the relationship between acoustic frequency of consonant sounds and magnitude-related meanings would ideally include fricatives, which are characterized by high-frequency noise. However, the extreme scarcity of fricatives in Australian languages prevents us from extending the set of associations we test in our sample in this way.

Regarding the more general debate about the universality of magnitude symbolism, the upshot of this study of Australian phonological patterns is clearer. There is a fair amount of variation in the specific sound/meaning patterns we find evidence for in individual languages. However, we also find at least one pattern of frequency-magnitude correspondence in more than half of the languages sampled and the significant distributional patterns we find across these languages are, in general, quite consistent with the expected sound symbolic relationships. This suggests that though the specific categories of sounds and magnitude-related meanings involved in sound symbolism may vary across languages, this variation exists within a more general pattern linking magnitude meanings to natural classes of speech sounds. These general trends cannot be explained by the genealogical relationships or geographic neighborhoods of the languages in which they occur, which leaves universal communicative function as a likely ultimate source for this sound-meaning relationship.

## Supporting Information

Figure S1 **Languages and forms used in the sound symbolism data set.**
(TXT)
